# Supplementary material for: Advanced-Retarded Differential Equations in Quantum Photonic Systems
Source: Sci Rep. 2017 Feb 23;7:42933. doi: 10.1038/srep42933 (PMC5322327; doi:10.1038/srep42933)
Supplement: Supplementary Information [file srep42933-s1.pdf]

Supplementary Information for "Advanced-Retarded Differential Equations in Quantum Photonic Systems"

Unai Alvarez-Rodriguez[1,*], Armando Perez-Leija[2,3], Iñigo L. Egusquiza[4], Markus Gräfe[2], Mikel Sanz[1], Lucas Lamata[1], Alexander Szameit[2,5], and Enrique Solano[1,6]

[1]Department of Physical Chemistry, University of the Basque Country UPV/EHU, Apartado 644, 48080 Bilbao, Spain
[2]Institute of Applied Physics, Abbe Center of Photonics, Friedrich-Schiller-Universität Jena, Max-Wien-Platz 1, Jena 07743, Germany
[3]Max Born Institute, Max Born Strasse 2A, 12489 Berlin, Germany
[4]Department of Theoretical Physics and History of Science, University of the Basque Country UPV/EHU, Apartado 644, 48080 Bilbao, Spain
[5]Institute for Physics, University of Rostock, Albert-Einstein-Straße 23, 18059 Rostock, Germany
[6]IKERBASQUE, Basque Foundation for Science, Maria Diaz de Haro 3, 48013 Bilbao, Spain
[*]Corresponding author: unaialvarezr@gmail.com

ABSTRACT

This document provides supplementary information to Advanced-Retarded Differential Equations in Quantum Photonic Systems. In particular, we provide examples of the genuine physical situation developed in the manuscript each of them associated with a given A.-R. equation, which show the versatility of our analog photonics simulator.

Non-Autonomous Equations

Consider the oscillatory time dependence of the propagation constant, β, for the single variable A-R equation, Eq. (1) in the manuscript. We numerically simulate this system for a lattice of $N = 6$ waveguides, $\beta = 1$, $\kappa = \sqrt{7}$, $\varepsilon = 1$ and $\omega = 2$, see Fig. 1. We have selected this non-autonomous advanced-retarded equation to show the existence resonant solutions, in which a high amount of light gets trapped in the chip. Notice that although a high population is achieved in the stationary state the theoretical error is still small.

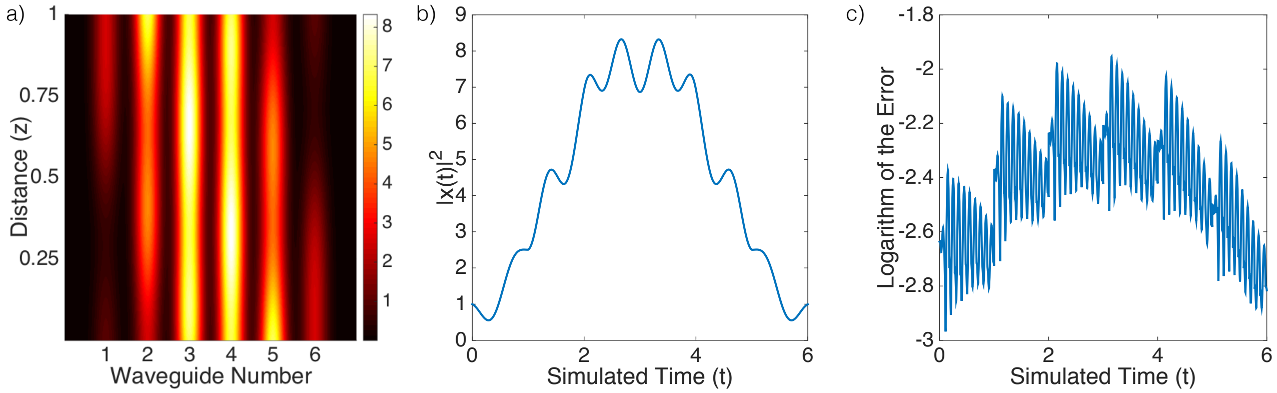

Figure 1. Supplementary Figure 1. Numerical Simulation of Eq. (1) with $N = 6$, $\beta = 1$, $\varepsilon = 1$, $\kappa = \sqrt{7}$, $\omega = 2$. (a) Intensity in the stationary state in the waveguides array. (b) Modulus square of the solution as a function of time. (c) Decimal Logarithm of the error of the simulation with respect to the solution of the A-R equation.

Systems of Equations

In Fig. 2 we show a numerical simulation of the array proposed to simulate systems of A-R equations given by Eq. (3). The dynamical parameters are the following ones, $N = 5$, $\beta_x = 1$, $\beta_y = 2$, $\kappa_x = 3$, $\kappa_y = 1$, $q = 1$, $d = 1$ and $\tau = 1$ for an initial state $|\psi(0)\rangle = |0\rangle$. The theoretical error is smaller than 10^{-2} for the complete time evolution. We have selected this parameters to show that highly asymmetric solutions are also possible for time independent equations even with the limitation induced by the physical constrains in the coupling constants κ.

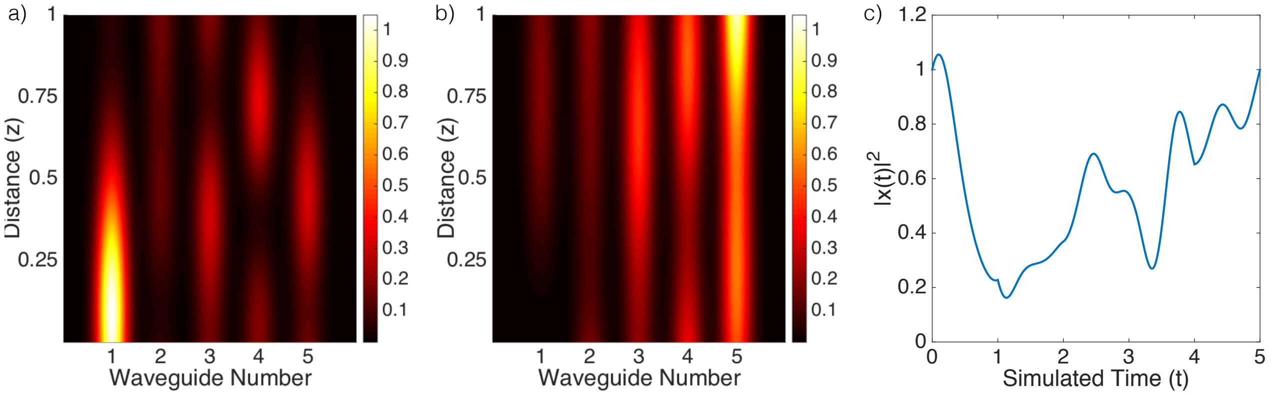

Figure 2. Supplementary Figure 2. Numerical Simulation of Eq. (3) with $N = 5$, $\beta_x = 1$, $\beta_y = 2$, $\kappa_x = 3$, $\kappa_y = 1$, $q = 1$, $d = 1$, $\tau = 1$ for the initial state $|\psi(0)\rangle = |0\rangle$. (a) Waveguides intensity in the x plane corresponding to the first component of the qubit. (b) Waveguides intensity in the y plane corresponding to the second component of the qubit. (c) Modulus square of the quantum state as a function of time.

Multiple Delays

Eq. (6) in the manuscript describes the evolution of a system driven by two feedback and two forward terms. See Fig. 3 for a numerical simulation of this equation with $N = 5$, $\beta = 1$, $\kappa = 5$ and $\tau = 1$.

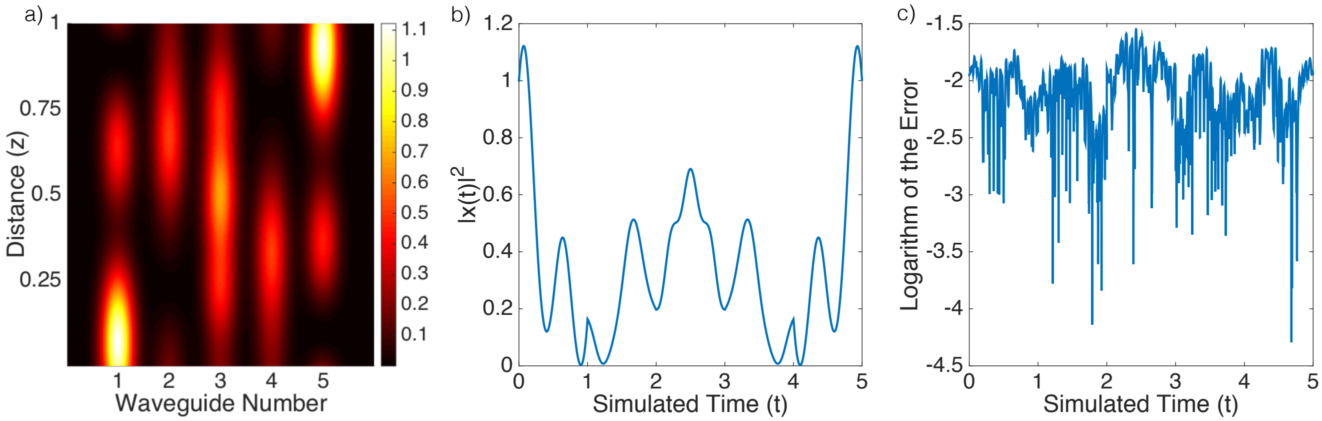

Figure 3. Supplementary Figure 3. Numerical simulation of Eq. (6) with $N = 5$, $\beta = 1$, $\kappa = 5$ and $\tau = 1$. (a) Intensity in the stationary state in the waveguides array. (b) Modulus square of the solution as a function of time. (c) Decimal Logarithm of the error of the simulation with respect to the solution of the A-R equation.
